# Genetic complexity impacts the clinical outcome of follicular lymphoma patients

**DOI:** 10.1038/s41408-020-00395-y

**Published:** 2021-01-11

**Authors:** María García-Álvarez, Sara Alonso-Álvarez, Isabel Prieto-Conde, Cristina Jiménez, M. Eugenia Sarasquete, M. Carmen Chillón, Alejandro Medina, Ana Balanzategui, Rebeca Maldonado, Alicia Antón, Marta Rodríguez, Oscar Blanco, Luis G. Díaz, Pilar Tamayo, Pedro Blanco, Carmen Esteban, Verónica González-Calle, Noemí Puig, Norma Gutiérrez, Alejandro Martín, Ramón García-Sanz, Marcos González, M. Dolores Caballero, Miguel Alcoceba

**Affiliations:** 1 Department of Hematology, University Hospital of Salamanca (HUS/IBSAL), CIBERONC and Cancer Research Institute of Salamanca-IBMCC (USAL-CSIC), Salamanca, Spain; 2 Department of Hematology, Central University Hospital of Asturias (HUCA), Oviedo, Spain; 3 Department of Pathology, University Hospital of Salamanca (HUS/IBSAL), Salamanca, Spain; 4 Department of Nuclear Medicine, University Hospital of Salamanca (HUS/IBSAL), Salamanca, Spain; 5 Department of Otorhinolaryngology, University Hospital of Salamanca (HUS/IBSAL), Salamanca, Spain; 6 Department of General and Gastrointestinal Surgery, University Hospital of Salamanca (HUS/IBSAL), Salamanca, Spain

**Keywords:** B-cell lymphoma, Genetics research

## Dear Editor,

Follicular lymphoma (FL) is the second most common non-Hodgkin lymphoma (NHL, 20–30%) after diffuse large B-cell lymphoma (DLBCL). Despite the introduction of rituximab and the high response rate to first-line treatment, approximately 20% of the FL patients relapse or progress within 2 years of receiving first-line therapy. Therefore, the major challenge is finding biomarkers that identify high-risk patients at diagnosis.

Qu et al. reported an association between increased genomic complexity, a concept based on copy-number aberrations (CNAs) and copy neutral loss of heterozygosity (cnLOH), and poor outcome in FL [1]. However, the concept of genetic complexity based on the number of mutated genes has not been analyzed in FL patients yet.

The aim of the present study was to analyze in detail the genetic landscape and genetic complexity by next-generation sequencing (NGS), defined by the number of mutated genes, in a FL series to improve our understanding of the biology of FL and its impact on the clinical outcome of the patients. See Supplementary Materials for details of methods.

A total of 83 FL grade I–IIIA patients diagnosed at the University Hospital of Salamanca between January 2000 and December 2017 were retrospectively included. The study was approved by the local Ethical Committee, in accordance with Spanish law and the Declaration of Helsinki. Written informed consent was obtained from all the patients. Clinical characteristics of the cohort are described in Table 1.

**Table 1:** Clinical characteristics of FL patients (*n* = 83).

Variable	Training cohort *n* (%)
Age, years (median, range)	62.5 (19–86)
Sex F/M	46/37
Histological grade^a^	
1	30 (38.0)
2	40 (50.6)
3A	9 (11.4)
FLIPI^a^	
0-1 (Low risk)	24 (32.0)
2 (Intermediate risk)	17 (22.7)
3-5 (High risk)	34 (45.3)
Ann Arbor^a^	
I	10 (14.1)
II	9 (12.7)
III	7 (9.9)
IV	45 (63.4)
First-line therapy	
Never treated	10 (12.0)
Palliative care	4 (4.8)
Rituximab-based ICT	47 (56.6)
R-CHOP	33 (39.8)
R-Bendamustine	3 (3.6)
R-CVP	3 (3.6)
R-FC	4 (4.8)
R-Lenalidomide	4 (4.8)
CT without rituximab	13 (15.7)
CHOP	11 (13.3)
Other	2 (2.4)
Radiotherapy alone or with rituximab	6 (7.2)
Rituximab alone	3 (3.6)
Maintenance with rituximab^a^	37 (53.6)
Response after induction therapy^a^	
CR	37 (53.6)
PR	28 (40.6)
NR/failure	4 (5.8)
CR30^a^	33 (47.8)

^a^Histological grade was available for 79 (95%) patients; FLIPI was available for 75 (90%) patients; Ann Arbor was available for 71 (86%) patients; Maintenance, response and CR30 were calculated for 69 (83%) patients. *CHOP* cyclophosphamide, doxorubicin, vincristine, prednisone; *CR* complete response, *CR30* complete response 30 months after the date induction treatment began, *CT* chemotherapy, *CVP* cyclophosphamide, vincristine, prednisone; *FLIPI* FL International Prognosis Index, *ICT* immunochemotherapy, *NR* no response, *PR* partial response, *R* rituximab.

We explored in patients who received rituximab-based immunochemotherapy (R-ICT, *n* = 47) the influence of clinical-biological variables at diagnosis on the following clinical endpoints: (i) complete response rate at 30 months (CR30) [2], defined as complete response 30 months after the first day of induction treatment; (ii) failure-free survival (FFS), defined as less than a partial response at the end of induction, relapse, progression, or death [3]; and (iii) overall survival (OS), defined as the time from the date of diagnosis of FL to that of death from any cause.

We identified 56 mutated genes in our FL series and 548 somatic nonsynonymous variants. FL patients harbored a median of 5 mutated genes (range 1–12) and 6 mutations (range 1–18) per case in our series. Fourteen genes were mutated with a frequency greater than 10%: *CREBBP* 63.9% (*n* = 53), *KMT2D/MLL2* 55.4% (*n* = 46), *BCL2* 41% (*n* = 34), *TNFRSF14* 27.7% (*n* = 23), *EZH2* 22.9% (*n* = 19), *STAT6* 19.3% (*n* = 12), *ARID1A* 18.1% (*n* = 15), *FOXO1* 18.1% (*n* = 15), *CARD11* 14.5% (*n* = 12), *EP300* 14.5% (*n* = 12), *GNA13* 13.2% (*n* = 11), *IRF8* 12% (*n* = 10), *SMARCA4* 12% (*n* = 10), and *HIST1H1E* 10.8% (*n* = 9) (Supplementary Fig. S1), being a subset of mutations clustered at known domains or hotspots (Supplementary Fig. S2).

The clinical impact of the 14 mutated genes with frequency >10% were explored. We first compared the clinical characteristics at diagnosis between groups to avoid potential biases in the analyses. *CREBBP* mutations were more frequently observed in younger FL patients (<60 years, *P* < 0.05). No other differences were observed for individual gene mutations regarding clinical characteristics of patients.

Regarding the clinical endpoints, mutations in *EZH2* were significantly associated with the presence of CR at 30 months (100% vs. 53.3%, *P* <0.05). Considering FFS, those FL patients with *FOXO1* mutations (25% vs. 68%, *P* <0.05) showed significantly shorter FFS at 5 years than the remaining patients. No other gene mutations were associated with CR30, FFS, or OS at 5 years.

All seven genes included in the m7-FLIPI clinical-genetic risk model described by Pastore et al. [3] were targeted by our NGS design, and therefore this model could be applied in 74 patients (89%). This score reclassified 17 high-risk FLIPI patients (23%) into the low-risk group. In our series, lower number of cases in CR30 (33% vs. 71%, *P* > 0.05), shorter FFS at 5 years (36% vs. 74%, *P* > 0.1), and shorter OS at 5 years (64% vs. 82%, *P* > 0.1) were found in high-risk m7-FLIPI patients although the differences were not statistically significant (Supplementary Fig. S3). In this series, FLIPI index was able to distinguish two groups with different OS at 5 years (69% vs. 88%, *P* < 0.05) (Supplementary Fig. S3).

In relation to genetic complexity, we observed a continuous FFS decrease as the number of mutated genes increased. Thus, we identified two groups with different FFS: 53 patients (63.9%) with ≤5 mutated genes and 30 patients (36.1%) with >5 mutated genes, which exactly matches with the median number of mutated genes and the result of the ROC analysis.

These two groups did not show significant differences in clinical characteristics including histological grade, FLIPI, and treatment requirement among others (Supplementary Table S1). By contrast, mutated genes showed a different distribution in both groups, underlining the high frequency of *BCL2* (63% vs. 28%, *P* < 0.01), *TNFRSF14* (43% vs. 19%, *P* < 0.05), *ARID1A* (30% vs. 11%, *P* < 0.05), *FOXO1* (33% vs. 9%, *P* < 0.05), *GNA13* (27% vs. 6%, *P* < 0.05), *IRF8* (27% vs. 4%, *P* < 0.01), and *MEF2B* (23% vs. 0%, *P* < 0.01) genes in group with >5 mutated genes.

**Fig. 1:**
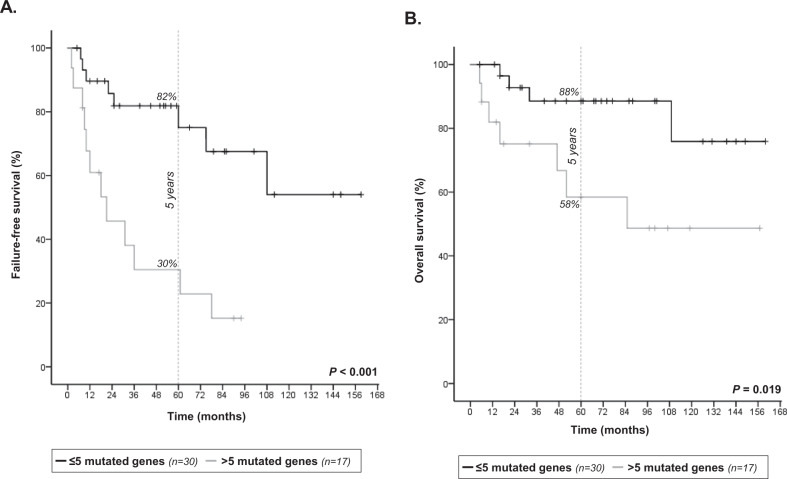
Genetic complexity and FL clinical outcome. Kaplan-Meier analysis of (**A**) failure-free survival and (**B**) overall survival by number of mutated genes in FL patients treated with R-ICT (*n* = 47). The vertical dashed line indicates 5-year follow-up.

A strong association was observed between >5 mutated genes and a lower number of cases in CR at 30 months as compared to ≤5 mutated genes (29% vs. 83%, *P* < 0.01). Moreover, patients with >5 mutated genes had a higher risk of treatment failure, since these patients showed shorter FFS at 5 years than those with ≤5 mutated genes (30% vs. 82%, *P* < 0.001) (Fig. 1A). Finally, patients with >5 mutated genes displayed shorter OS at 5 years than patients with ≤5 mutated genes (58% vs. 88%, *P* < 0.05) (Fig. 1B).

In the multivariate analysis, including FLIPI and m7-FLIPI as co-variables besides those statistically significant in univariate analysis, the only variable independently associated with shorter FFS was having >5 mutated genes (HR: 5.5, 95% CI: 2.1–14.6) (Supplementary Table S2). In terms of OS, to present >5 mutated genes (HR: 5.4, 95% CI: 1.5–19.6) and high-risk FLIPI (HR: 7.2, 95% CI: 1.3–39.1) at the time of diagnosis were independently associated with shorter OS (Supplementary Table S2).

In the present study, the mutational landscape analysis has allowed to identify 14 highly recurring mutated genes considered as drivers or early events in FL pathogenesis, which are consistent with previous reports [4–7]. In addition, we evaluated the m7-FLIPI clinicogenetic risk model in a real-world cohort of 47 R-ICT-treated patients, allowing the reclassification of 50% high-risk FLIPI patients in low-risk group, similar to the original series [3]. The m7-FLIPI showed no statistically significant differences in terms of FFS or OS between high-risk and low-risk patients in our series, probably due to the size of study cohort. However, the FLIPI index distinguished groups with different OS in this series. Moreover, in line with our results, other studies have also found no association of m7-FLIPI with different outcomes in real-world and clinical trial populations [5, 8].

In this work, we showed for the first time the association of genetic complexity, defined by the number of mutated genes, and FL outcome, independently of FLIPI. Genetic complexity in our study was defined as >5 mutated genes among 66 and it was associated with a poor outcome assessed by low number of cases in CR at 30 months, poor FFS, and short OS. In a very recent work, an increased genomic complexity defined by the number of CNAs was associated with worse FFS and OS in FL [1]. The impact of the number of mutations or the number of CNAs in the clinical course of patients has been reported in other hematological malignancies [9, 10].

Even associated to genetic complexity, we pointed out the association of *FOXO1* mutations with adverse outcome. *FOXO1* mutations had already been correlated to worse FFS in FL [3], and worse OS in DLBCL [11], and they were found at high prevalence in relapsed/refractory FL [12], thus supporting its adverse role in FL outcome. Finally, *EZH2* mutations were associated to the persistence of complete response after 30 months from therapy initiation (CR30), although they were equally distributed between genetic complexity groups, thus suggesting a subgroup of patients with better prognosis independently of the genetic complexity. This is in line with prior studies that associate *EZH2* mutations with favorable prognosis in FL [13–15].

In summary, our study has increased the knowledge of the FL biology through the characterization of the mutational landscape of a representative series of FL patients, and allowed to confirm the dependence of the tumor germinal center B-cell on alterations in genes involved in epigenetic modification, signaling, and transcription factors. Our results identified genetic alterations associated with prognosis in a cohort treated with chemoimmunotherapy based on rituximab. The most outstanding result, however, was that genetic complexity related with lower number of CR30 cases, worse FFS, and shortened OS, independently of FLIPI. Although the small number of patients could limit some analysis and further validation is required, we have shown that genetic complexity constitutes a promising robust tool that may be incorporated into risk predictive models, and may improve risk classification of FL to help in the development of new risk-adapted therapies.

## Supplementary information

Supplementary Materials

Supplementary Figures
