# Supplementary material for: Genetic complexity impacts the clinical outcome of follicular lymphoma patients
Source: Blood Cancer J. 2021 Jan 11;11(1):11. doi: 10.1038/s41408-020-00395-y (PMC7801414; doi:10.1038/s41408-020-00395-y)
Supplement: Supplementary file 1 — Supplementary Materials [file 41408_2020_395_MOESM1_ESM.pdf]

TITLE

GENETIC COMPLEXITY IMPACTS THE CLINICAL OUTCOME OF FOLLICULAR LYMPHOMA PATIENTS.

RUNNING TITLE

Genetic Complexity Influences the FL Clinical Course.

AUTHORS

María García-Álvarez,[1] Sara Alonso-Álvarez,[2] Isabel Prieto-Conde,[1] Cristina Jiménez,[1] M. Eugenia Sarasquete,[1] M. Carmen Chillón,[1] Alejandro Medina,[1] Ana Balanzategui,[1] Rebeca Maldonado,[1] Alicia Antón,[1] Marta Rodríguez,[3] Oscar Blanco,[3] Luis G. Díaz,[4] Pilar Tamayo,[4] Pedro Blanco,[5] Carmen Esteban,[6] Verónica González-Calle,[1] Noemí Puig,[1] Norma Gutiérrez,[1] Alejandro Martín,[1] Ramón García-Sanz,[1] Marcos González,[1] M. Dolores Caballero,[1] Miguel Alcoceba.[1]

AFFILIATIONS

1) Department of Hematology, University Hospital of Salamanca (HUS/IBSAL), CIBERONC and Cancer Research Institute of Salamanca-IBMCC (USAL-CSIC), Salamanca, Spain

2) Department of Hematology, Central University Hospital of Asturias (HUCA), Oviedo, Spain

3) Department of Pathology, University Hospital of Salamanca (HUS/IBSAL), Spain

4) Department of Nuclear Medicine, University Hospital of Salamanca (HUS/IBSAL), Spain

5) Department of Otorhinolaryngology, University Hospital of Salamanca (HUS/IBSAL), Spain

6) Department of General and Gastrointestinal Surgery, University Hospital of Salamanca (HUS/IBSAL), Spain.

CORRESPONDENCE TO: Dr. RAMÓN GARCÍA-SANZ

Servicio de Hematología
Hospital Universitario de Salamanca
Pº San Vicente 58-182
37007-Salamanca
Spain
TELEPHONE: +34 923 291384
FAX: +34 923 294624
E-MAIL: rgarcias@usal.es

CONTENTS:

1. **Supplementary Subjects and Methods**
2. **Supplementary Tables**
3. **Supplementary Figure legends**

1. SUPPLEMENTARY SUBJECTS AND METHODS

Exclusion criteria

FL grade IIIB and composite cases of FL+DLBCL were not included. Cases without biological sample at diagnosis, or those with poor quality and/or insufficient DNA were also not included. Formalin-fixed paraffin-embedded (FFPE) samples instead of fresh tissue were used in cases with low tumor cell infiltration (<10%) by flow cytometry.

DNA Extraction

Genomic DNA (gDNA) was extracted from 160 biopsies corresponding to tumor (n=83) and matched non-tumor (n=77) biological samples. gDNA from fresh-frozen tissue (38.1%) was isolated by phenol-chloroform method or the Maxwell® 16 System (Promega, Madison, WI, USA). In cases where gDNA was extracted from bone marrow (28.2%), peripheral blood (24.3%) and other tissues (1.9%), the Maxwell® 16 System (Promega) or DNAzol reagent (MRC, Cincinnati, OH, USA) were used. Finally, gDNA from FFPE tissue (7.5%) was isolated using the RecoverAll Total Nucleic Acid Isolation Kit (Ambion/Applied Biosystems, Foster City, CA, USA) or the QIAamp DNA FFPE Tissue Kit (Qiagen, Hilden, Germany).

DNA was quantified by Qubit® 2.0 Fluorometer (Invitrogen™, Eugene, OR, USA) using the dsDNA BR (Broad-Range) Assay kit. The quality and purity of the gDNA extracted from FFPE tissue were assessed with the 4200 TapeStation (Agilent Technologies, Santa Clara, CA, USA) system using the Genomic DNA ScreenTape assay.

Library Preparation and Next Generation Sequencing

Samples were purified using Genomic DNA clean & Concentrator TM-10 (Zymo Research, Irvine, California, USA). Generation of libraries was carried out by two different technologies. Libraries from twenty-one (25.3%) patients were obtained using a TruSeq Custom Amplicon v1.5 gene panel (Illumina Inc., San Diego, CA, USA) targeted at coding regions (CDS) of 56 genes, which was designed by Illumina DesignStudio platform. Libraries from remaining 62 patients (74.7%) were generated with an improved target enrichment SeqCap EZ Choice gene panel (Roche NimbleGen Inc., Madison, WI) that including the CDS of 66 genes (see below), which was designed by NimbleDesign platform and allowed to sequence all FFPE samples included in the study. The resulting pool in each panel was normalized and combined (twelve-plex) before undergoing high-throughput paired-end (201 bp and 101 bp, respectively) sequencing. Sequencing was performed on an Illumina MiSeq System with 1000-fold ± 500-fold coverage for samples from any origin. In 77/83 cases (92.8%), matched non-tumor DNA from bone marrow and peripheral blood was sequenced to exclude germ-line variants. In the remaining 6 cases, matched non-tumor DNA could not be obtained because of early death.

Sequence alignment and variant calling was performed using MiSeq Reporter software (Illumina Inc.) and data visualization was made using Integrative Genomics Viewer (IGV). Annotation of resulting variant call files (.vcfs) was performed with BaseSpace Variant Interpreter software, filtering out SNVs and small insertions/deletions with <100X reads and variant allele frequency (VAF) of <5%. Variants in the non-coding regions, synonymous variants, single nucleotide polymorphism (SNPs, MAF>1%) and platform-specific artefacts were also excluded.

>> <u>Genes included in both custom panels:</u> *ARID1A, B2M, BCL2, BCL6, BRAF, CARD11, CCND3, CD70, CD79A, CD79B, CDKN2A, CDKN2B, CREBBP, CXCR4, DTX1, EBF1, EP300, EZH2, FBXO11, FOXO1, GNA13, GNAI2, HIST1H1B, HIST1H1C, HIST1H1D, HIST1H1E, ID3, IRF8, KRAS, MAML1, MCL1, MEF2B, MLL2/KMT2D, MYC, MYD88, NOTCH1, NOTCH2, NRAS, P2RY8, PAX5, PIM1, POU2F2/OCT2, PRDM1, RHOA, RPS15, RRAGC, S1PR2, SMARCA4, SPEN, TBL1XR1, TCF3, TMSB4X, TNFAIP3, TNFRSF14, TP53, XPO1*.

>> Genes unique to the enrichment SeqCap EZ Choice gene panel: *CD58, CIITA, FAS, KLHL6, PDL1/CD274, SGK1, SOCS1, STAT3, STAT6, TMEM30A.*

Detection of t(14;18)/*IGH-BCL2*

Interphase fluorescence in situ hybridization (FISH) analysis was carried out to detect t(14;18)/*IGH-BCL2* by using the Vysis LSI *IGH-BCL2* Dual Color, Dual Fusion Translocation probe (Abbott Molecular, Des Plaines, IL, USA) following manufacturer's recommendations. The presence of the t(14;18)/*IGH-BCL2* rearrangement was also analyzed by nested PCR with an adapted method from Gribben *et al*. using two BCL2 primers targeting the major breakpoint region (MBR) and minor cluster region (mcr) in chromosome 18 and a consensus primer against JH region in chromosome 14. The JH internal primer was labelled with a fluorochrome to detect the BCL2 translocation size by GeneScan with an ABI 3500xL DNA Sequencer (Applied Biosystems).

Statistical analysis

Associations between variables were analyzed by the χ^2-square test for categorical variables, and by Student's unpaired-samples t-test or the Mann-Whitney U test for continuous variables. Cut-off values for relevant continuous variables were estimated using ROC curves. Survival was estimated by the Kaplan–Meier method and differences were assessed by the log-rank test. Subsequently, all variables for which there was some indication of a significant association with clinical features in the univariate test (P<0.1) were considered in a multivariate Cox regression model. Differences were considered to be statistically significant for values of P<0.05. All analyses were performed using SPSS (IBM SPSS 23.0, IBM Corp, Armonk, NY, USA).

2. <u>SUPPLEMENTARY TABLES</u>

Supplementary Table S1. Clinical and biological characteristics of FL patients included according to number of mutated genes (≤5 mutated genes, n=53; >5 mutated genes, n=30).

Variable	≤5 mutated genes, n (%)	>5 mutated genes, n (%)	*P*
**Median follow-up, months (range)**	72.5 (5-236)	70 (6-220)	*ns*
**Age ≥60 years**	25 (47.2)	20 (69.0)	*ns*
**Sex F/M**	28 (53) / 25 (47)	18 (60) / 12 (40)	*ns*
**Histological grade***			*ns*
1	22 (43.1)	8 (28.6)	
2	24 (47.1)	16 (57.1)	
3A	5 (9.8)	4 (14.3)	
**FLIPI***			*ns*
0-1 (Low risk)	15 (31.3)	9 (33.3)	
2 (Intermediate risk)	11 (22.9)	6 (22.2)	
3-5 (High risk)	22 (45.8)	12 (44.4)	
**Ann Arbor***			*ns*
I	6 (13.0)	4 (16.0)	
II	4 (8.7)	5 (20.0)	
III	2 (4.3)	5 (20.0)	
IV	34 (73.9)	11 (44.0)	
**t(14;18) present**	42 (79.2)	27 (90.0)	*ns*
**First-line therapy**			*ns*
Never treated	6 (11.3)	4 (13.3)	
Palliative care	2 (3.8)	2 (6.7)	
Rituximab-based ICT	30 (56.6)	17 (56.7)	
R-CHOP	22 (73.3)	11 (64.7)	
R-Bendamustine	2 (6.7)	1 (5.9)	
R-CVP	1 (3.3)	2 (11.8)	
R-FC	3 (10.0)	1 (5.9)	
R-Lenalidomide	2 (6.7)	2 (11.8)	
CT without rituximab	10 (18.9)	3 (10.0)	
CHOP	9 (90.0)	2 (66.7)	
Other	1 (10.0)	1 (33.3)	
Radiotherapy alone or with rituximab	3 (5.7)	3 (10.0)	
Rituximab alone	2 (3.8)	1 (3.3)	
**Maintenance with rituximab***	25 (83.0)	12 (71.0)	*ns*

*≤5 mutated genes: Histological grade was available for 51 (96%) patients; FLIPI was available for 48 (91%) patients; Ann Arbor was available for 46 (87%) patients; Maintenance calculated for 30 (57%) patients.

>5 mutated genes: Histological grade was available for 28 (93%) patients; FLIPI was available for 27 (90%) patients; Ann Arbor was available for 25 (83%) patients; Maintenance calculated for 17 (57%) patients.

CHOP, cyclophosphamide, doxorrubicin, vincristine, prednisone; CVP, cyclophosphamide, vincristine, prednisone; FLIPI, FL International Prognosis Index; ICT, immunochemotherapy; CT, chemotherapy; R, rituximab.

Supplementary Table S2. Univariate and multivariate analysis of factors influencing failure-free survival (FFS) and overall survival (OS) in rituximab-based ICT patients (n=47).

Variable	n	FFS (%) 5-years	U (*P*-value)	M (*P*-value)	HR [CI 95%]
**FLIPI**					
**Low/Intermediate** *(reference)*	21	74.1%	0.119	-	-
**High**	25	55.5%		0.143	2.3 [0.7-7.4]
**m7-FLIPI**					
**Low** *(reference)*	34	73.9%	0.112	-	-
**High**	12	36.5%		0.495	1.5 [0.5-4.7]
**Mutated genes**					
**Five or less** *(reference)*	30	81.9%	$4.1x10^{-4}$	-	-
**More than five**	16	30.5%		0.001	5.5 [2.1-14.6]
FOXO1					
**Wild-type** *(reference)*	42	68.1%	0.027	-	-
**Mutated**	4	25.0%		0.075	3.3 [0.9-12.7]
**Variable**	**n**	**OS (%) 5-years**	**U (P-value)**	**M (P-value)**	**HR [CI 95%]**
**FLIPI**					
**Low/Intermediate** *(reference)*	21	88.2%	0.021	-	-
**High**	25	68.8%		0.021	7.2 [1.3-39.1]
**m7-FLIPI**					
**Low** *(reference)*	34	82.5%	0.242	-	-
**High**	12	64.3%		0.798	1.2 [0.3-4.5]
**Mutated genes**					
**Five or less** *(reference)*	30	88.5%	0.019	-	-
**More than five**	17	58.4%		0.011	5.4 [1.5-19.6]

FLIPI, Follicular Lymphoma International Prognostic Index; HR, hazard ratio; CI, confidence interval; M, multivariate analysis; OS, overall survival; FFS, failure free survival; U, univariate analysis.

3. <u>SUPPLEMENTARY FIGURE LEGENDS</u>

Supplementary Figure S1. Genetic landscape of FL. Mutation plot for 83 patients shows the 56 genes with somatic nonsynonymous variants in our FL series. Columns represent each patient and are ordered by the number of mutated genes, represented by a color gradient. Genes are clustered based on functional category. The bar graph on the left corresponds to the percentage of cases with mutations in that gene. The presence of t(14;18) is represented at the bottom. Asterisk indicates genes not included in amplicon design.

Supplementary Figure S2. Mutation distribution across the genes with a mutation frequency greater than 10%. *CREBBP* mutations were principally located in the histone acetyltransferase (HAT) domain or next to this domain. *TNFRSF14* mutations truncated the extracellular domain of protein, grouping in exons 3 and 4, fundamentally. *EZH2* mutations affected the Y646 residue, which is a hotspot located in C-terminal methyltransferase SET domain, and *STAT6* mutations were mainly clustered in D419 residue which is part of DNA binding domain. In the case of *FOXO1*, missense mutations were located mainly in exon 1. *EP300* mutations affected HAT domain, and *CARD11* mutations were mostly found in the Coiled-coil domain of scaffold protein. The remaining genes (*KMT2D*, *BCL2*, *ARID1A*, *GNA13*, *IRF8*, *SMARCA4* and *HIST1H1E*) harbored disruptive mutations distributed across the whole gene.

Supplementary Figure S3. Kaplan-Meier analysis of (A) failure-free survival and overall survival by m7-FLIPI model, and (B) overall survival by FLIPI index in FL patients treated with R-ICT (n=47). The vertical dashed line indicates 5-year follow-up.
