# Supplementary material for: Genetic complexity impacts the clinical outcome of follicular lymphoma patients
Source: Blood Cancer J. 2021 Jan 11;11(1):11. doi: 10.1038/s41408-020-00395-y (PMC7801414; doi:10.1038/s41408-020-00395-y)

Supplementary Figure S1. Genetic landscape of FL.

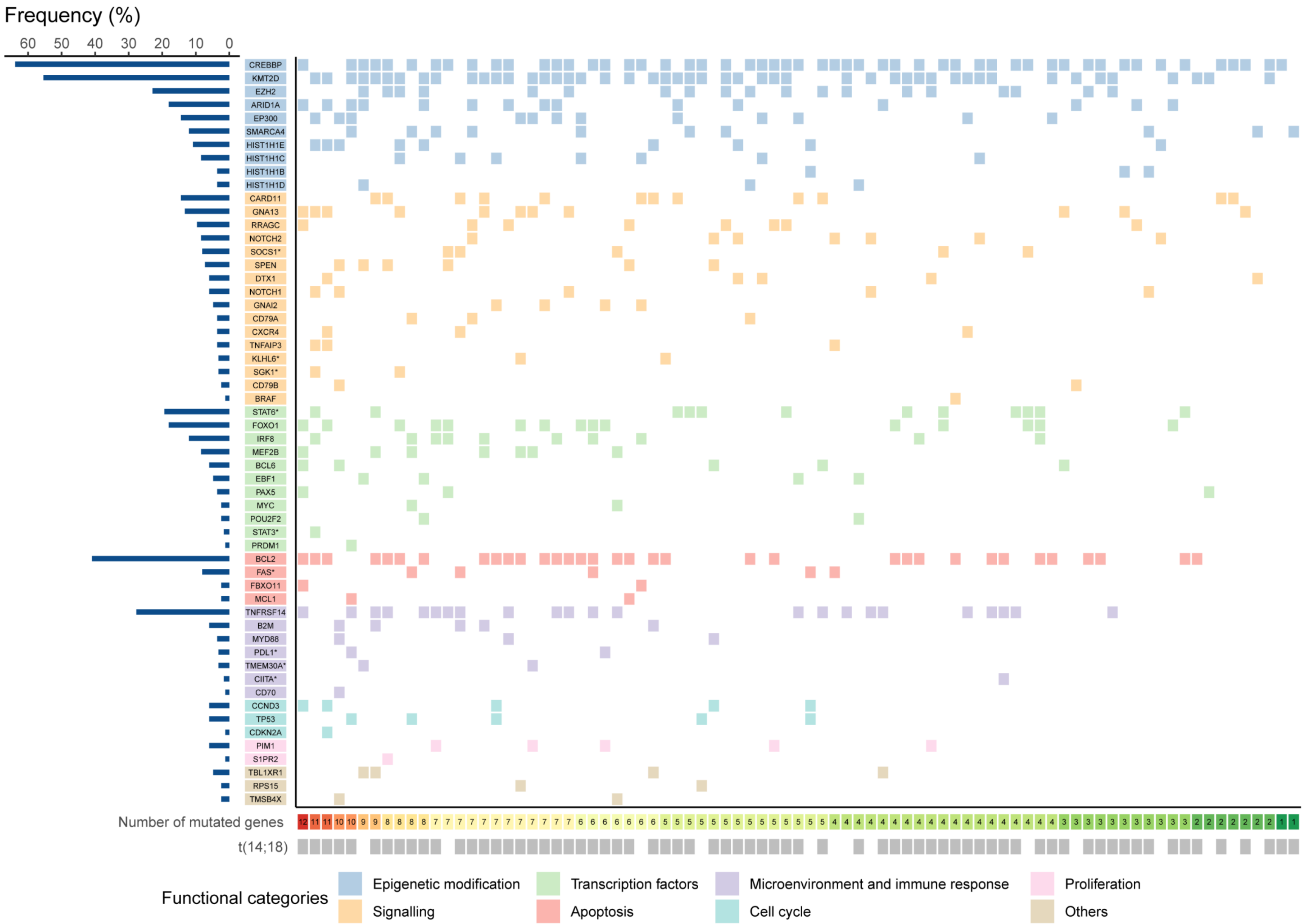

Supplementary Figure S2. Mutation distribution across the genes with a mutation frequency greater than 10%.

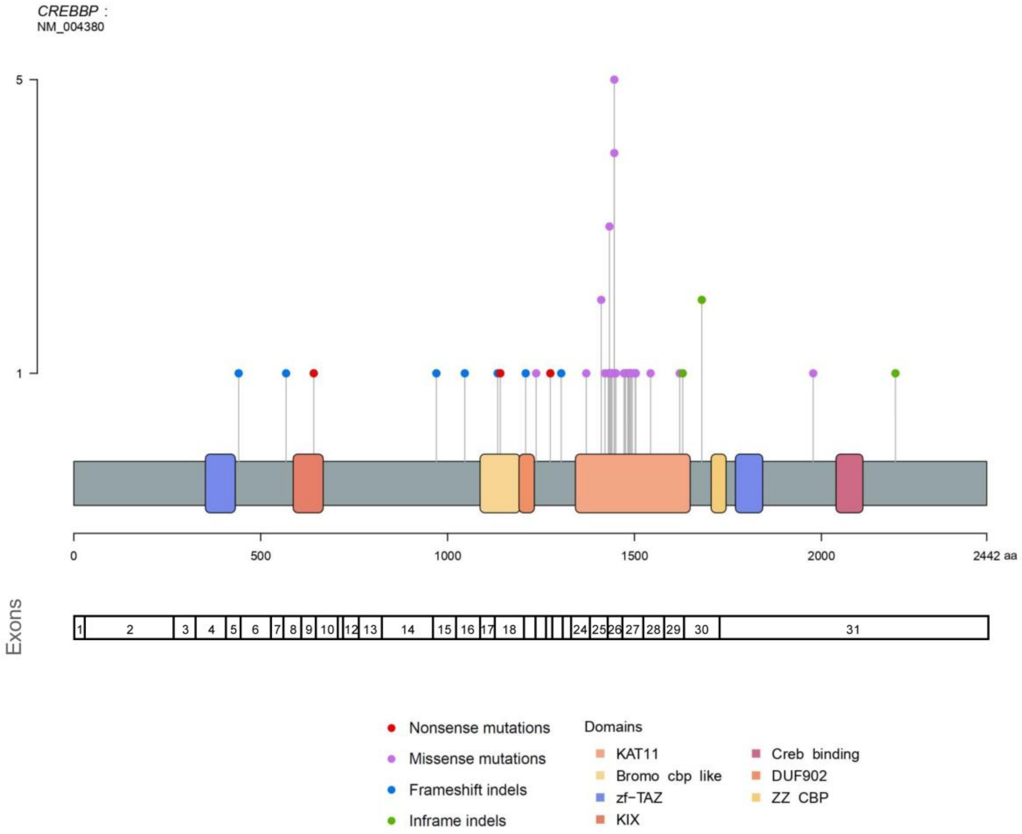

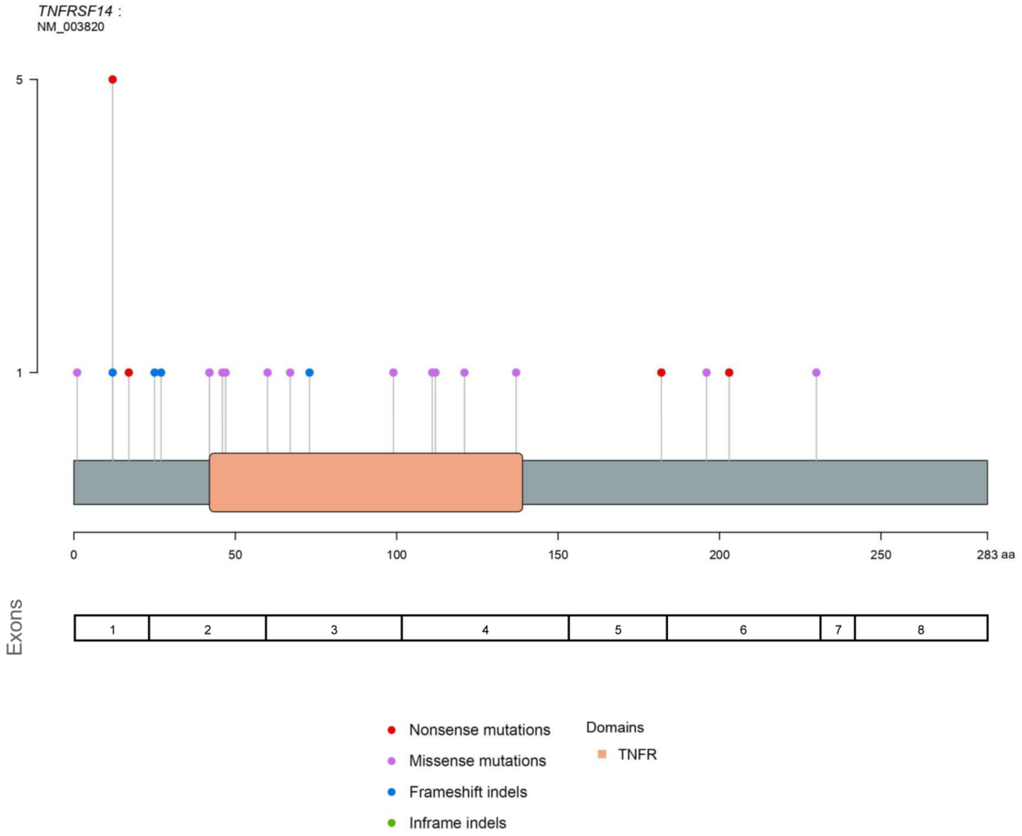

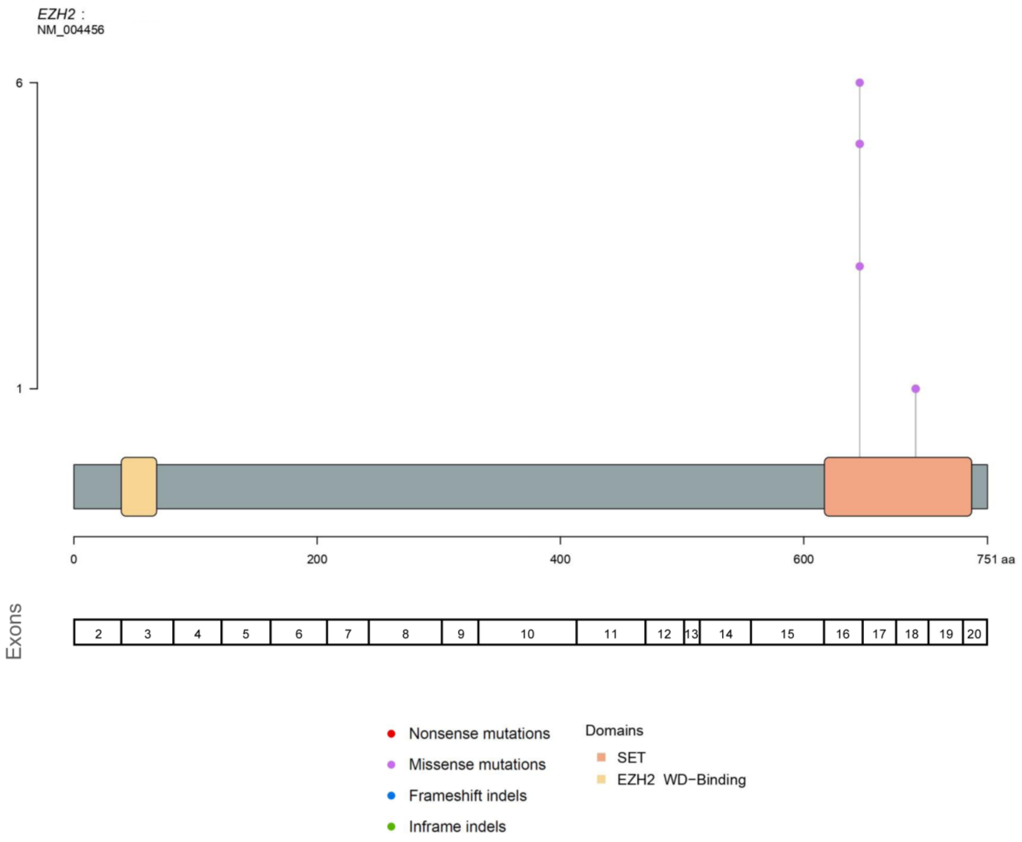
EZH2 :
NM_004456
6
1
0
200
400
600
751 aa
Exons
2 3 4 5 6 7 8 9 10 11 12 13 14 15 16 17 18 19 20
Nonsense mutations
Missense mutations
Frameshift indels
Inframe indels
Domains
SET
EZH2 WD−Binding

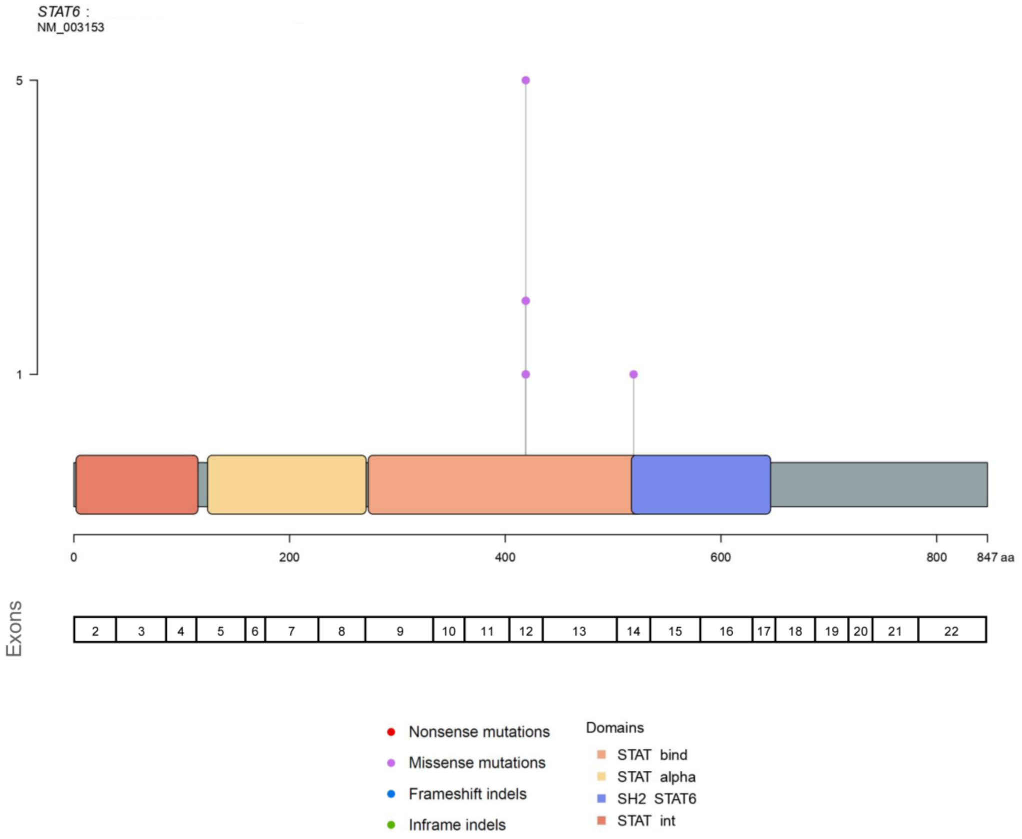
STAT6 :
NM_003153
5
1
0
200
400
600
800
847 aa
Exons
2 3 4 5 6 7 8 9 10 11 12 13 14 15 16 17 18 19 20 21 22
Nonsense mutations
Missense mutations
Frameshift indels
Inframe indels
Domains
STAT bind
STAT alpha
SH2 STAT6
STAT int

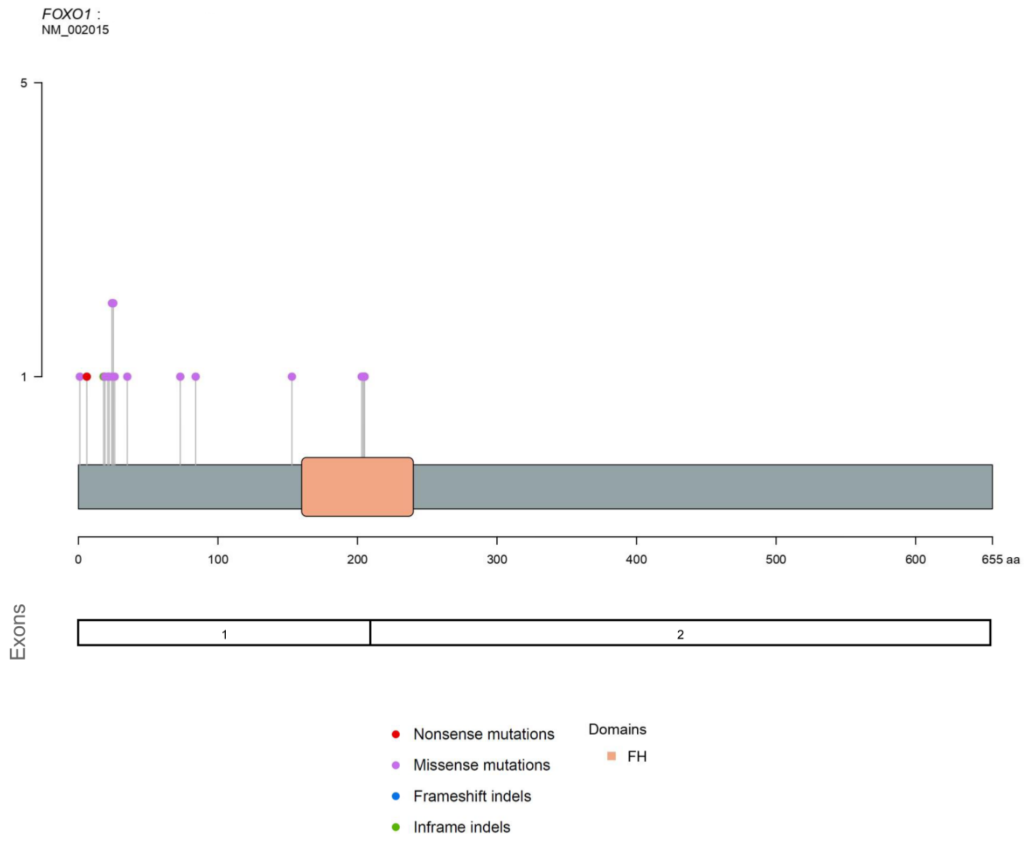
FOXO1 :
NM_002015
5
1
0
100
200
300
400
500
600
655 aa
Exons
1
2
Nonsense mutations
Missense mutations
Frameshift indels
Inframe indels
Domains
FH

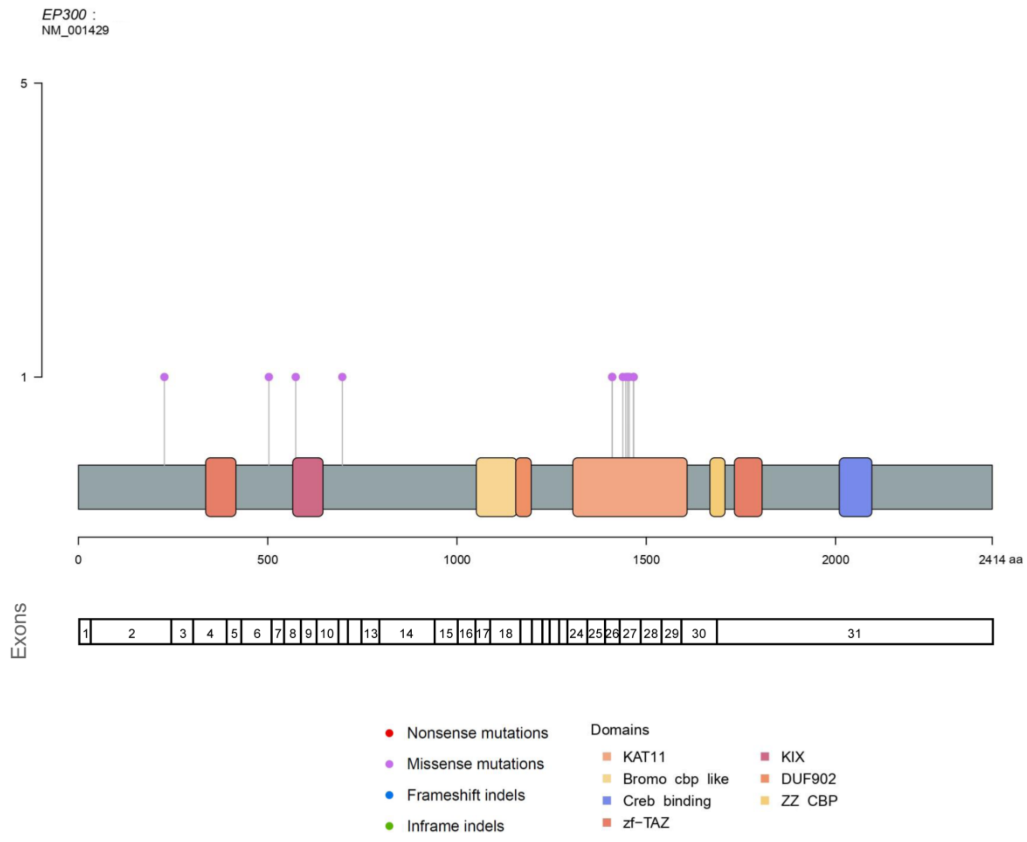
EP300 :
NM_001429
5
1
0
500
1000
1500
2000
2414 aa
Exons
1
2
3
4
5
6
7
8
9
10
13
14
15
16
17
18
24
25
26
27
28
29
30
31
Nonsense mutations
Missense mutations
Frameshift indels
Inframe indels
Domains
KAT11
Bromo cbp like
Creb binding
zf−TAZ
KIX
DUF902
ZZ CBP

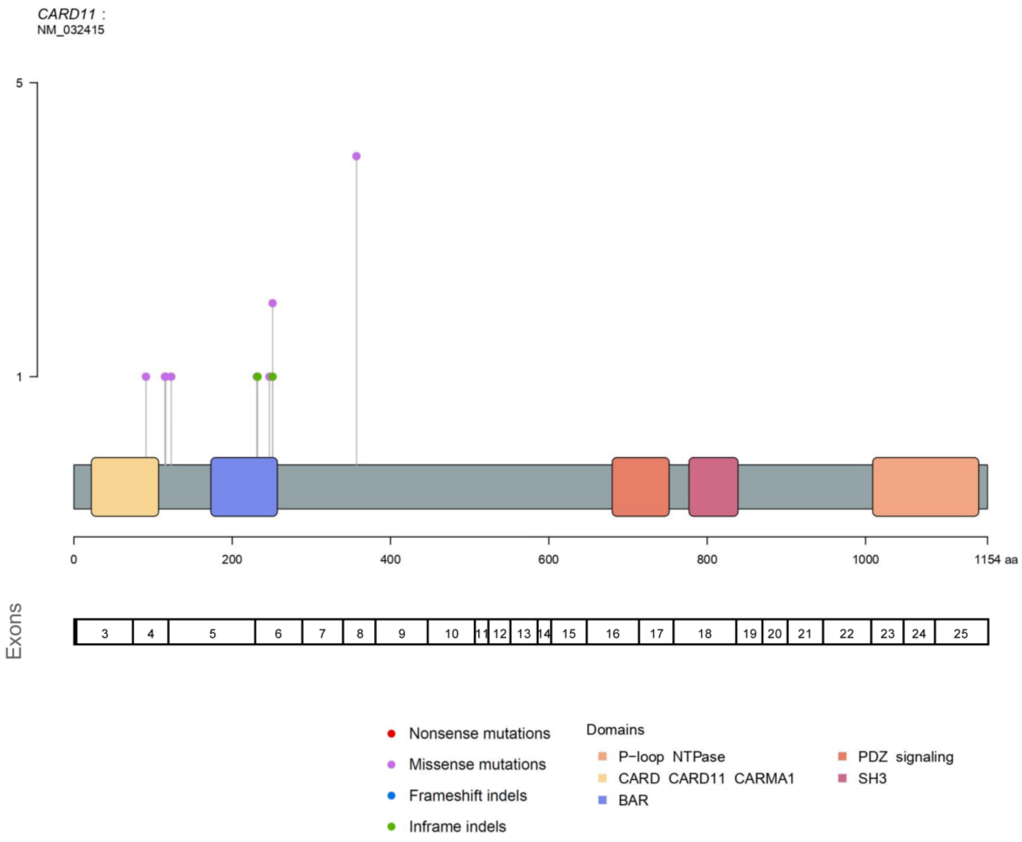
CARD11 :
NM_032415
5
1
0
200
400
600
800
1000
1154 aa
Exons
3 4 5 6 7 8 9 10 11 12 13 14 15 16 17 18 19 20 21 22 23 24 25
Nonsense mutations
Missense mutations
Frameshift indels
Inframe indels
Domains
P−loop NTPase
CARD CARD11 CARMA1
BAR
PDZ signaling
SH3

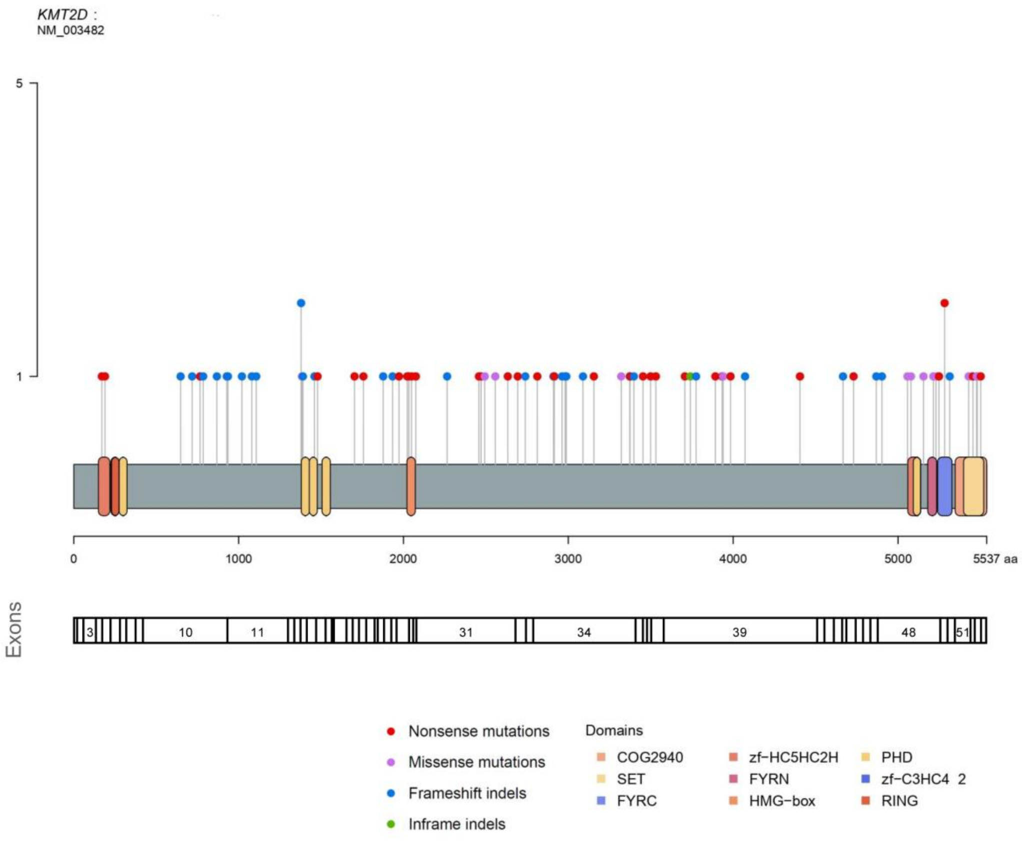
KMT2D :
NM_003482
5
1
0
1000
2000
3000
4000
5000
5537 aa
Exons
3 10 11 31 34 39 48 51
Nonsense mutations
Missense mutations
Frameshift indels
Inframe indels
Domains
COG2940
SET
FYRC
zf−HC5HC2H
FYRN
HMG−box
PHD
zf−C3HC4 2
RING

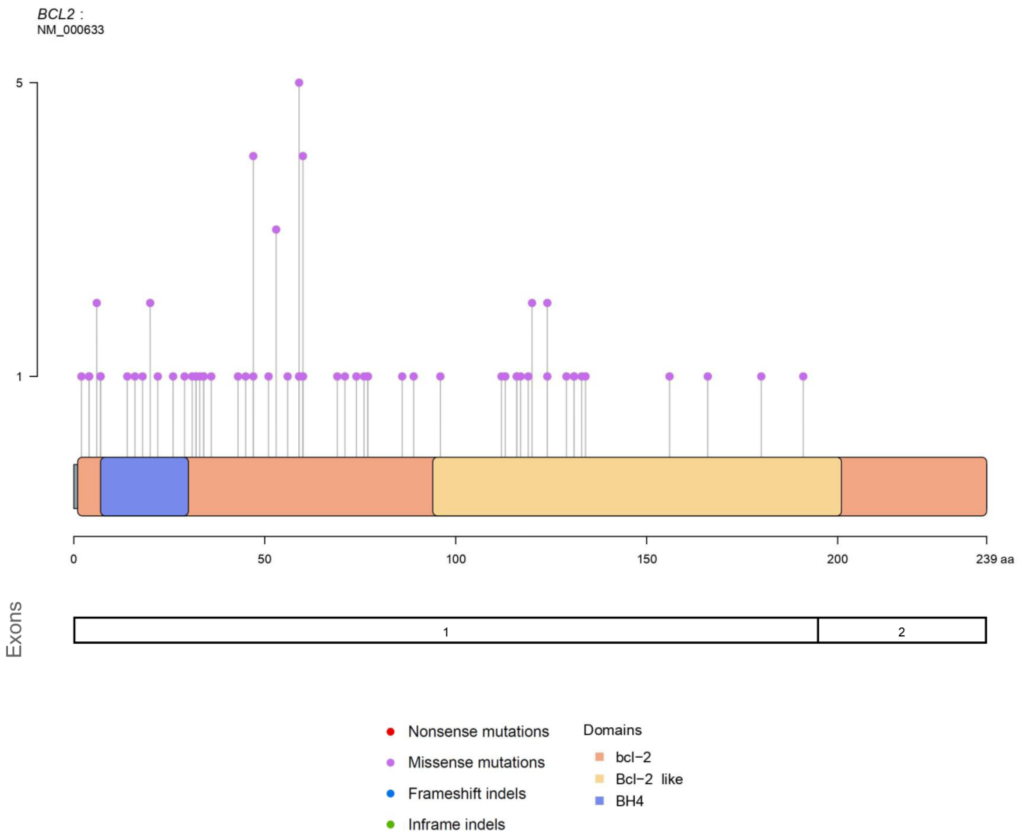
BCL2 :
NM_000633
5
1
0
50
100
150
200
239 aa
Exons
1
2
Nonsense mutations
Missense mutations
Frameshift indels
Inframe indels
Domains
bcl−2
Bcl−2 like
BH4

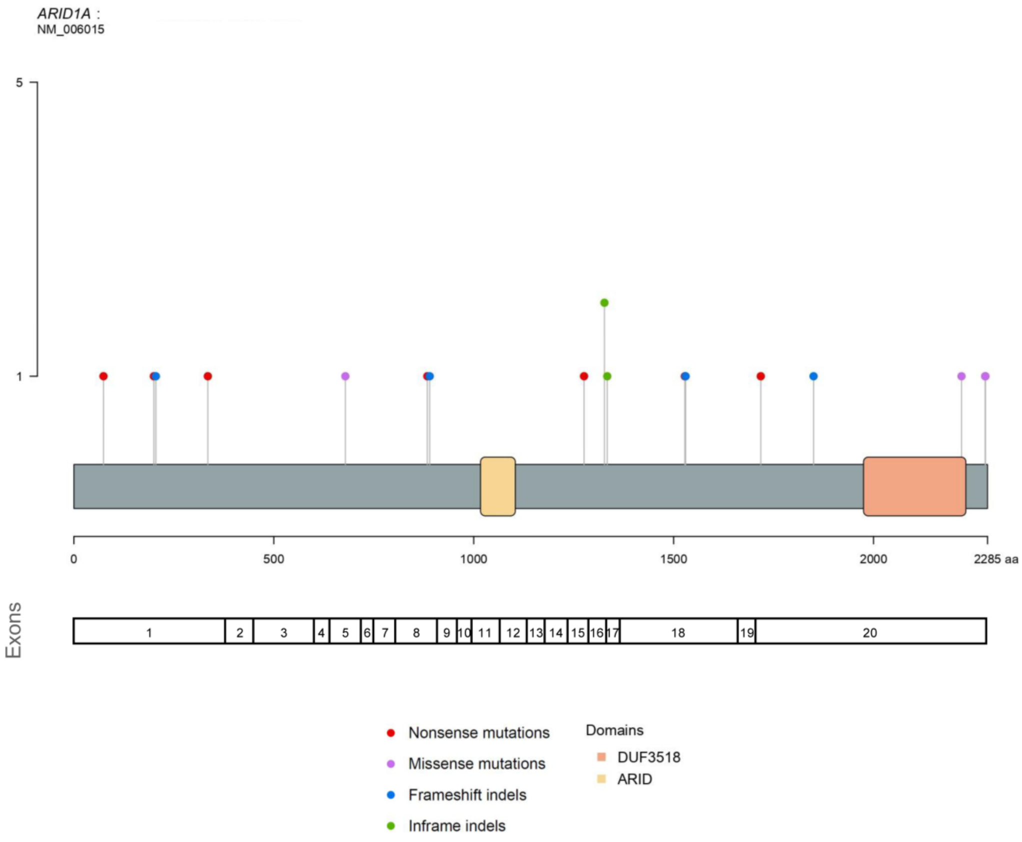
ARID1A :
NM_006015
5
1
0
500
1000
1500
2000
2285 aa
Exons
1
2
3
4
5
6
7
8
9
10
11
12
13
14
15
16
17
18
19
20
Nonsense mutations
Missense mutations
Frameshift indels
Inframe indels
Domains
DUF3518
ARID

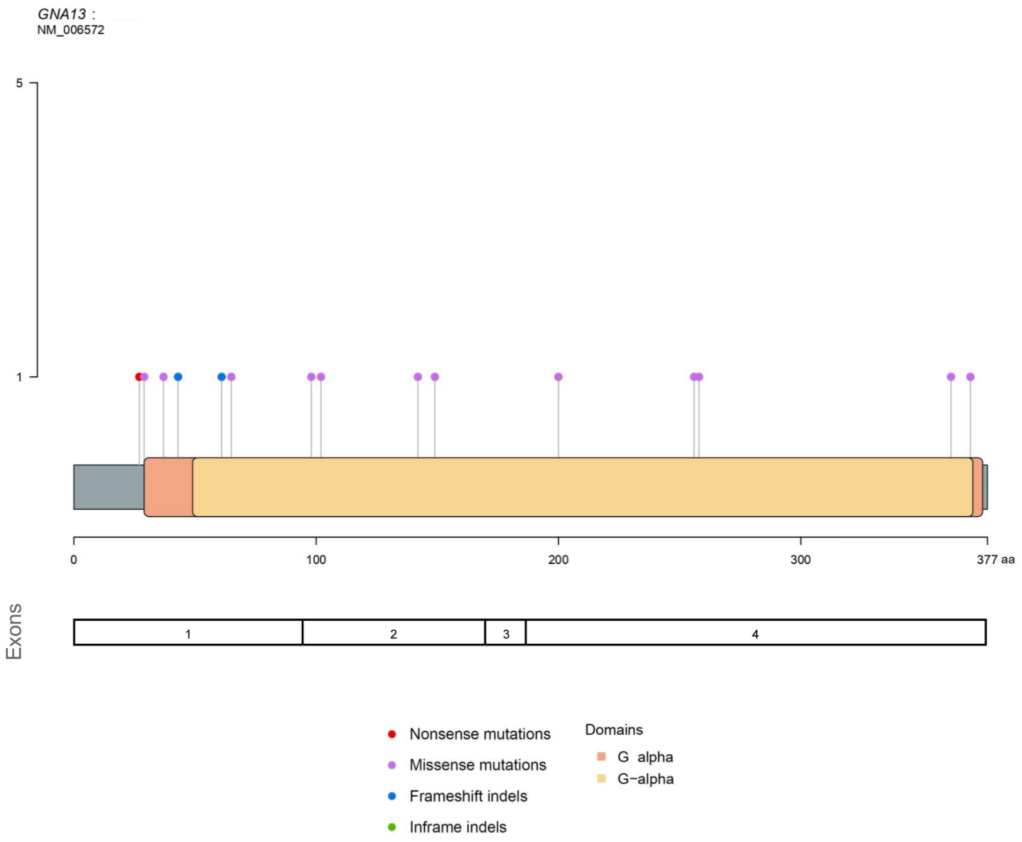

GNA13 :
NM_006572
5
1
0
100
200
300
377 aa
Exons
1
2
3
4
Nonsense mutations
Missense mutations
Frameshift indels
Inframe indels
Domains
G alpha
G−alpha

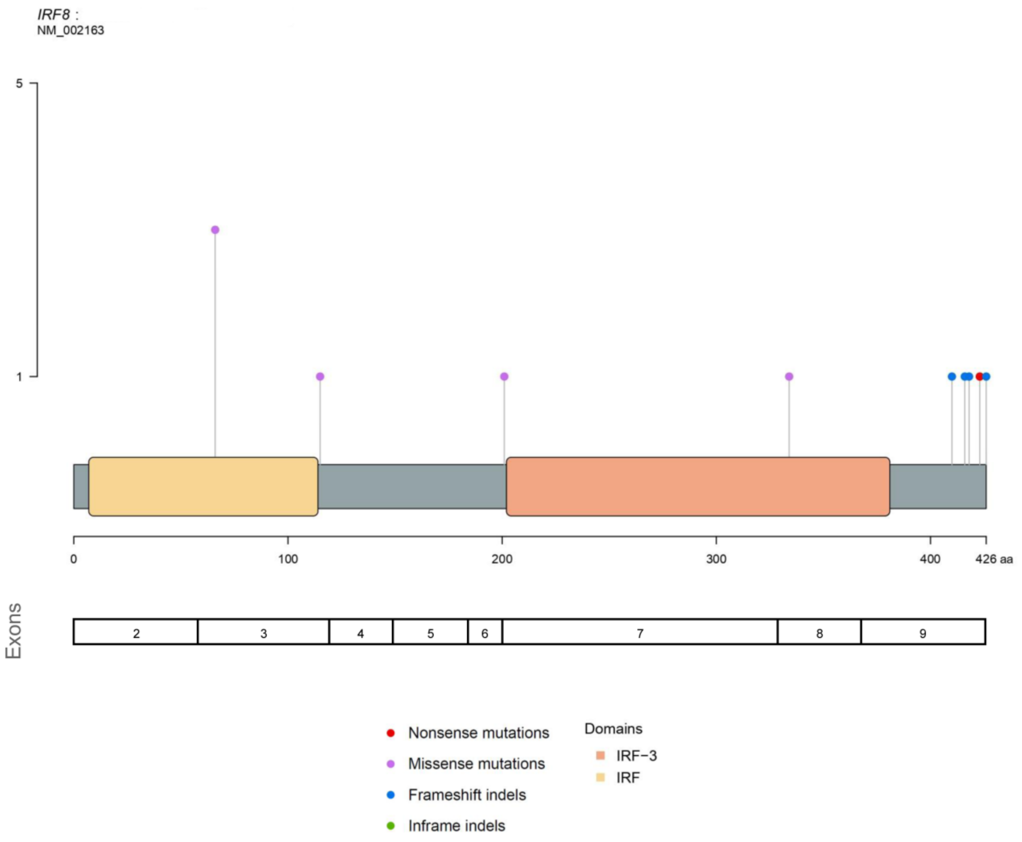

IRF8 :
NM_002163
5
1
0
100
200
300
400
426 aa
Exons
2
3
4
5
6
7
8
9
Nonsense mutations
Missense mutations
Frameshift indels
Inframe indels
Domains
IRF−3
IRF

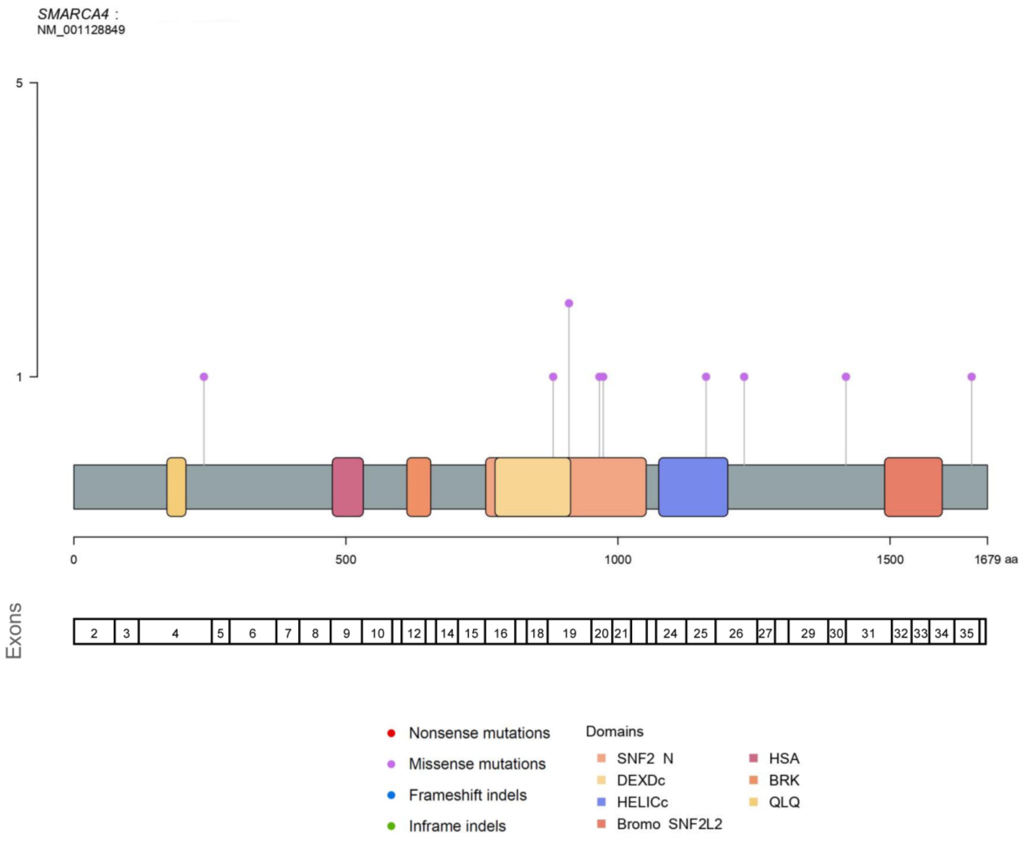
SMARCA4 :
NM_001128849
5
1
0
500
1000
1500
1679 aa
Exons
2 3 4 5 6 7 8 9 10 12 14 15 16 18 19 20 21 24 25 26 27 29 30 31 32 33 34 35
Nonsense mutations
Missense mutations
Frameshift indels
Inframe indels
Domains
SNF2 N
DEXDc
HELICc
Bromo SNF2L2
HSA
BRK
QLQ

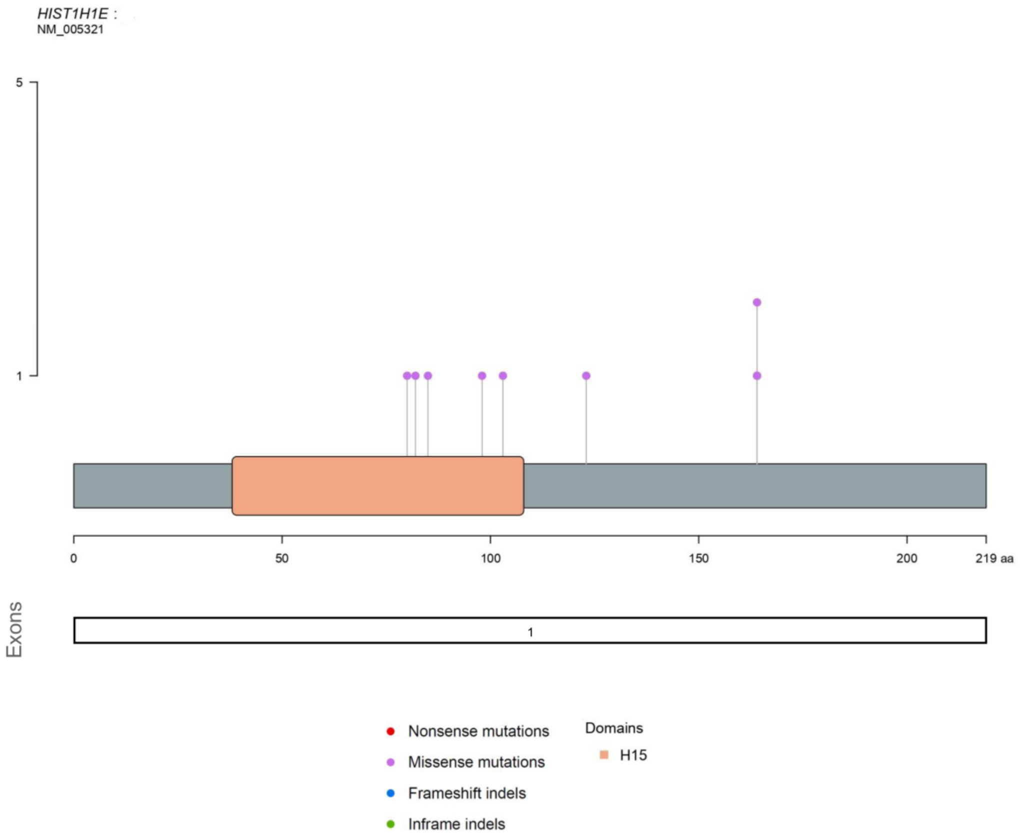
HIST1H1E :
NM_005321
5
1
0
50
100
150
200
219 aa
Exons
1
Nonsense mutations
Missense mutations
Frameshift indels
Inframe indels
Domains
H15

Supplementary Figure S3. Kaplan-Meier analysis of (A) failure-free survival and overall survival by m7-FLIPI model, and (B) overall survival by FLIPI index in FL patients treated with R-ICT (n=47). The vertical dashed line indicates 5-year follow-up.

A. m7-FLIPI

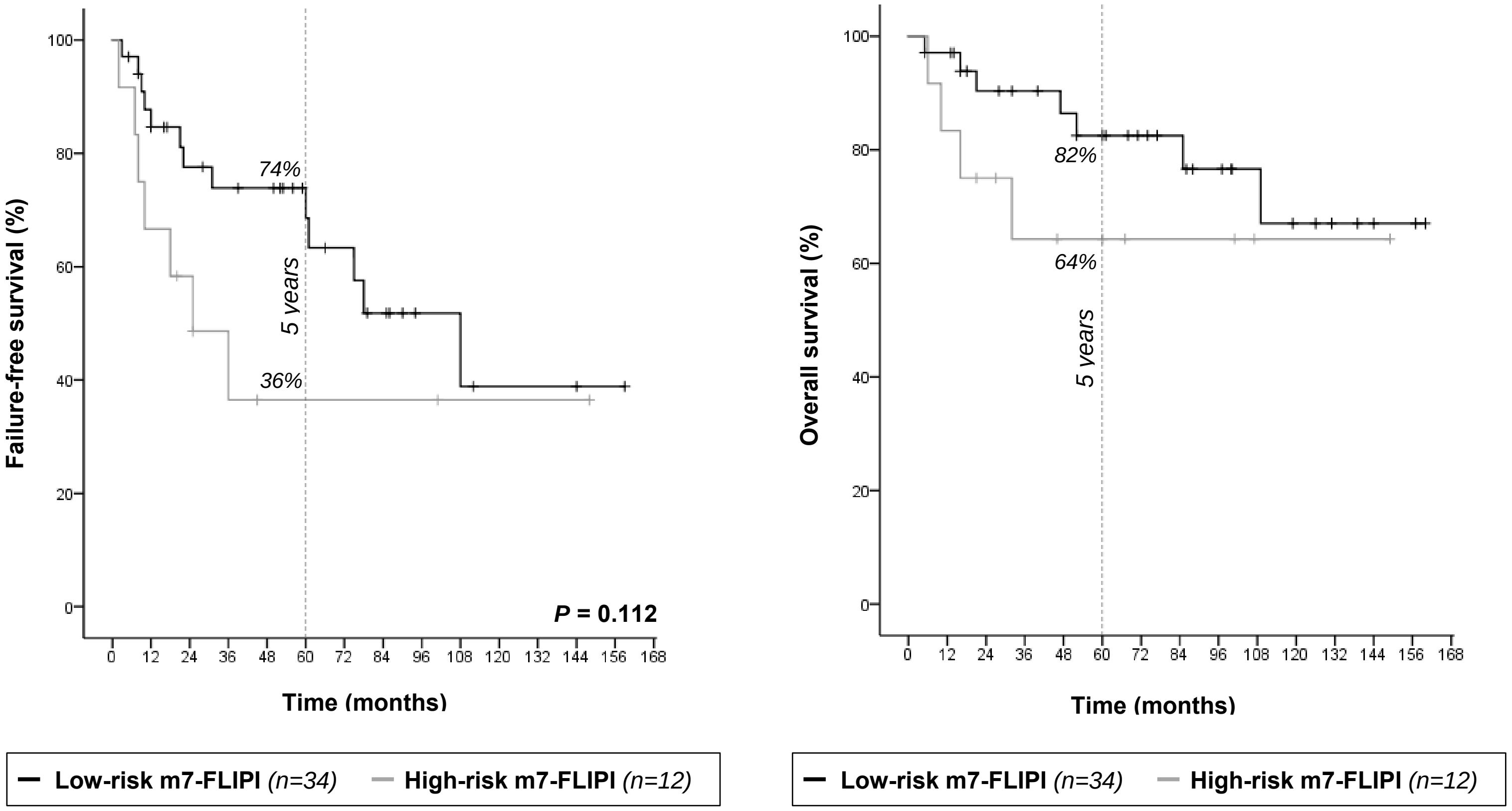

B. FLIPI

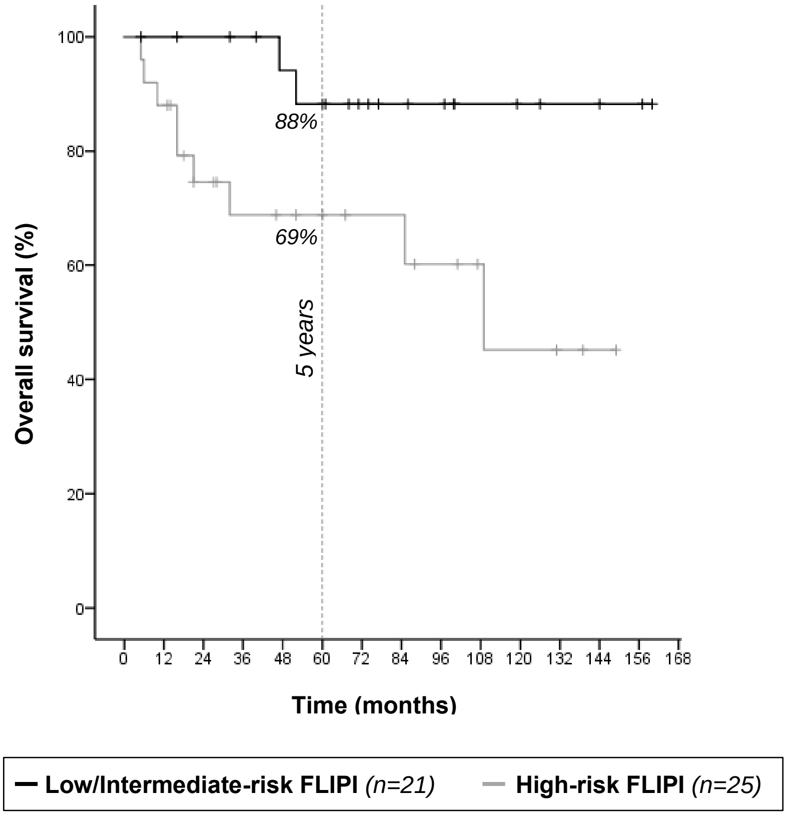

Supplement: Supplementary file 2 — Supplementary Figures [file 41408_2020_395_MOESM2_ESM.pdf]
